# An NMR Approach for Investigating Membrane Protein–Lipid Interactions Using Native Reverse Micelles

**DOI:** 10.21769/BioProtoc.5039

**Published:** 2024-07-20

**Authors:** Sara H. Walters, Brian Fuglestad

**Affiliations:** 1 Department of Chemistry, Virginia Commonwealth University, Richmond, VA, USA; 2 Institute for Structural Biology, Drug Discovery and Development, Virginia Commonwealth University, Richmond, VA, USA

**Keywords:** Membrane models, Peripheral membrane proteins, Protein NMR, Protein–membrane interactions, Reverse micelles

## Abstract

Peripheral membrane proteins (PMPs) are a subgroup of membrane-associated proteins that are water-soluble and bind to membranes, often reversibly, to perform their function. These proteins have been extensively studied in the aqueous state, but there is often a lack of high-resolution structural and functional studies of these proteins in the membrane-bound state. Currently, nuclear magnetic resonance (NMR) is among the best-equipped methods to study these relatively small proteins and domains, but current models have some disadvantages that prevent a full understanding of PMP interactions with membranes and lipids. Micelles, bicelles, and nanodiscs are all available for NMR observation but are based on synthetic lipids that may destabilize proteins or are too large to accommodate straightforward structural analysis. This protocol introduces a method for forming reverse micelles using lipids from natural sources, here called native reverse micelles. This technique allows the PMPs to embed within a shell of naturally derived lipids surrounding a small water core solubilized in an alkane solvent. PMP embedment in the lipid shell mimics binding to a cellular membrane. Here, naturally derived lipids from soy, bovine heart, and porcine brain are used in conjunction with n-dodecylphosphocholine (DPC) to encapsulate a PMP from either concentrated or dried protein, resulting in reverse micelles that may be confirmed via dynamic light scattering and NMR. This protocol allows for high-quality NMR data of PMPs interacting with membrane lipids within a biologically accurate environment.

Key features

• This protocol describes using natural lipids to construct reverse micelles for high-resolution NMR studies of proteins.

• Initial optimization of encapsulation conditions proceeds through visual assessment, with dynamic light scattering (DLS) to measure size distribution, and NMR to observe protein behavior.

• Membrane-interacting proteins are encapsulated in their membrane-bound state. Proteins that do not interact with membranes are housed in their water-solubilized state.

• Structural, functional, and inhibitory studies may be performed on native reverse micelle-encapsulated proteins.

## Graphical overview


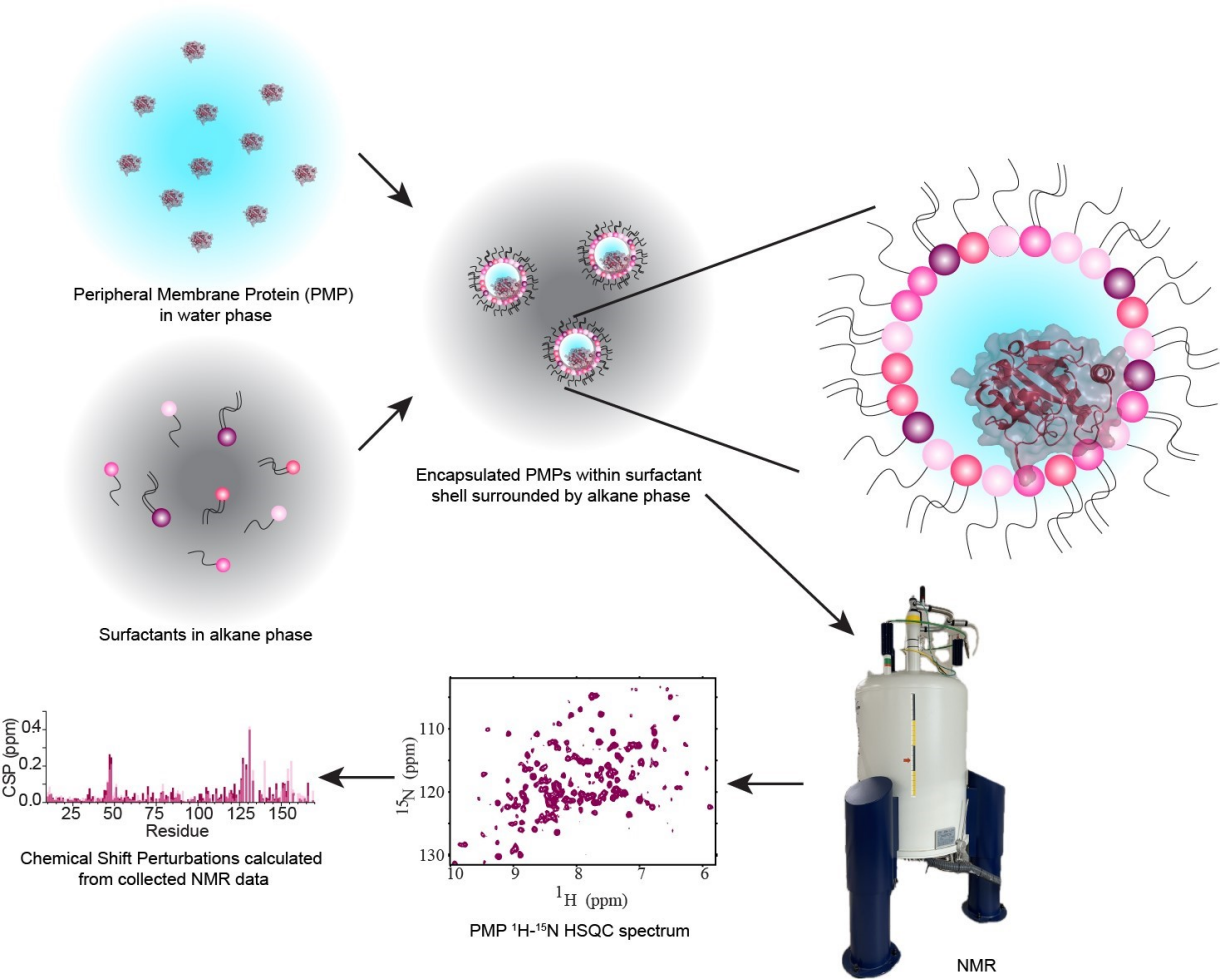


## Background

Peripheral membrane proteins (PMPs) are water-soluble proteins that can bind, often reversibly, with membranes. Interactions between PMPs and their target membranes are of great interest due to the central role of these proteins in disease and biology. Currently, methods to accurately observe the protein–membrane interaction, especially at high resolution, are limited. Nuclear magnetic resonance (NMR) is well-suited to observe these interactions using micelles, bicelles, and nanodiscs. However, these membrane models are most commonly formulated from artificial components, limiting understanding in a biological context. Reverse micelles (RMs) have a long history in the study of proteins, including being used to house membrane-associated and membrane-integral proteins for NMR [1,2]. RMs house proteins within a nanoscale pool of water, surrounded by a hollow shell of surfactants with the hydrophilic headgroups interacting with the water and the hydrophobic tails pointing outward and serving to solubilize the RM in an alkane solvent. High-quality NMR spectra of proteins encapsulated within RMs are routinely attainable, enabled by the low-viscosity alkane solvent [3]. Recent developments of RMs have improved their properties as membrane mimetics through formulations using phosphocholine-based surfactants. Utilizing membrane-mimicking RMs (mmRMs), the PMP of interest can be observed interacting with the surfactant shell. This allows the mapping of PMP-membrane interaction surfaces and enables structural and functional studies using NMR. RMs formulated with naturally derived lipids represent an advancement in technology, allowing the study of PMPs and other proteins in a more native-like membrane mimetic [4]. The lipid extractions used for native reverse micelles (nRMs), including soy lecithin, bovine heart lipids, and porcine brain lipids, account for a wide range of lipid compositions. These compositions introduce a heterogeneous mixture of lipid headgroup and tail types, reflecting the complexity of biological membranes. Conversely, a formulation of RMs made entirely from phosphocholine-based surfactants can be used as a standard background to explore specific lipid interactions or for structural and functional studies that require a more homogeneous membrane model [5]. Previous applications of RMs to protein studies may also be extended to nRMs, including structural determination of PMPs [6], hydration dynamics measurements [7], experimental cosolvent mapping [8], and enhanced fragment screening [9]. Integral membrane proteins and proteins anchored through lipidations have previously been housed in RMs and would benefit from study in the more native-like environment described here [1,2]. The following protocol describes the construction of reverse micelles from lipids extracted from native sources or phosphocholine-based surfactants, for high-resolution NMR study of encapsulated PMPs in their membrane-bound state.

## Materials and reagents

**Biological materials**

Protein of interest; in this protocol, glutathione peroxidase 4 (GPx4) is used as an example for encapsulation in reverse micelles. Protein was produced as previously described using recombinant ^15^N or ^13^C-^15^N isotopic labeling, expression, and purification from BL21(DE3) *E. coli* [10].

**Reagents**

1. Lecithin (VWR, catalog number: 10791-822)
2. Ethylenediaminetetraacetic acid (EDTA) (Fisher, catalog number: AAJ15694AE)
3. Sodium chloride (NaCl) (Fisher, catalog number: S271-1)
4. Bis-Tris (VWR, catalog number: 6976-37-0)
5. Tris base (Sigma-Aldrich, catalog number: 77-86-1)
6. Chloroform (Fisher, catalog number: 67-66-3)
7. Methanol (EM Science, catalog number: 67-56-1)
8. Distilled water
9. Hydrochloric acid (HCl) (Fisher, catalog number: SA49)
10. Dithiothreitol (DTT) (GoldBio, catalog number: DTT10)
11. Sodium sulfate (VWR, catalog number: 7757-82-6)
12. Brain total lipid extract (porcine) (PBL) (Avanti Polar Lipids, catalog number: 131101C)
13. Heart total extract (bovine) (BHL) (Avanti Polar Lipids, catalog number: 171201P)
14. Pentane (Alfa Aesar, catalog number: AA32449K2)
15. 1-Hexanol (Sigma-Aldrich, catalog number: 111-27-3)
16. 1,2-dilauroyl-sn-glycero-3-phosphocholine (DLPC) (Avanti Polar Lipids, catalog number: 850335C)
17. n-dodecylphosphocholine (DPC) (Avanti Polar Lipids, catalog number: 850336P)
18. D-pentane (Sigma-Aldrich, catalog number: 490482)
19. D_2_O (Sigma-Aldrich, catalog number: 7789-20-0)
20. Trimethyl phosphate (Sigma-Aldrich, catalog number: 512-56-1)
21. Bradford dye (Thermo Fisher, catalog number: 23238)

**Solutions**

1. 5 M NaCl stock solution (see Recipes)
2. 500 mM Bis-Tris pH 6.0 stock solution (see Recipes)
3. 1 M DTT stock solution (see Recipes)
4. 200 mM EDTA pH 6.0 stock solution (see Recipes)
5. 6 M HCl stock solution (see Recipes)
6. Protein buffer (see Recipes)
7. Dilute protein buffer (see Recipes)
8. Mobile phase for soy lecithin chelation (see Recipes)

**Recipes**

1. **5 M NaCl stock solution**
*Note: Initially, add half of the distilled H_2_O to dissolve the NaCl; the rest of the volume should slowly be added as NaCl dissolves. The stock should be brought up to the final volume once all NaCl is dissolved to account for any volume displacement caused by the solid powder.
  **Table:** 

Reagent	Final concentration	Quantity or Volume
NaCl	5 M	146.1 g
H_2_O	n/a	*see Note
Total	n/a	500 mL
2. **500 mM Bis-Tris pH 6.0 stock solution**
*Note: Initially, add half of the distilled H_2_O to dissolve the Bis-Tris; most of the rest should slowly be added as Bis-Tris dissolves. Once the Bis-Tris is dissolved, add small increments of 6 M HCl into the solution to bring the pH to 6.0. The stock should be brought up to the final volume with distilled H_2_O once the Bis-Tris is pH-corrected to account for any volume displacement caused by the solid powder and HCl.
  **Table:** 

Reagent	Final concentration	Quantity or Volume
Bis-Tris pH 6.0	500 mM	52.3 g
H_2_O	n/a	*see Note
pH corrected by HCl		*see Note
Total	n/a	500 mL
3. **1 M DTT stock solution**
*Note: Initially, add half of the distilled H_2_O to dissolve the DTT; the rest should slowly be added as DTT dissolves. The stock should be brought up to the final volume once all DTT is dissolved to account for any volume displacement caused by the solid powder.
  **Table:** 

Reagent	Final concentration	Quantity or Volume
DTT	1 M	1.54 g
H_2_O	n/a	*see Note
pH adjusted using HCl		
Total	n/a	10 mL
4. **200 mM EDTA pH 6.0 stock solution**
*Note: Initially, add half of the distilled H_2_O to dissolve the EDTA; most of the rest should slowly be added as EDTA dissolves. Once the EDTA is dissolved, add small increments of 6 M HCl to the solution to bring the pH to 6.0. The stock should be brought up to the final volume with distilled H_2_O once the EDTA is pH-corrected to account for any volume displacement caused by the solid powder and HCl.
  **Table:** 

Reagent	Final concentration	Quantity or Volume
EDTA	200 mM	2.92 g
H_2_O	n/a	*see Note
pH adjusted using HCl		
Total	n/a	50 mL
5. **6 M HCl stock solution**
  **Table:** 

Reagent	Final concentration	Quantity or Volume
HCl, 12 N	6 M	100 mL
H_2_O	n/a	100 mL
Total	6 M	200 mL
6. **Protein buffer**
  **Table:** 

Reagent	Final concentration	Quantity or Volume
Bis-Tris pH 6.0, 500 mM stock	20 mM	2 mL
NaCl, 5 M stock	100 mM	1 mL
DTT, 1 M stock	20 mM	1 mL
H_2_O	n/a	46 mL
Total	n/a	50 mL
7. **Dilute protein buffer**
  **Table:** 

Reagent	Final concentration	Quantity or Volume
Bis-Tris pH 6.0, 500 mM stock	20 μM	2.4 μL
NaCl, 5 M stock	100 μM	1.2 μL
DTT, 1 M stock	20 μM	1.2 μL
H_2_O	n/a	59.952 mL
Total	n/a	60 mL
8. **Mobile phase for soy lecithin chelation**
*Note: Dry the solvents needed with sodium sulfate overnight and decant before the mobile phase is made.*
  **Table:** 

Reagent	Final concentration	Quantity or Volume
Chloroform	n/a	10 mL
Methanol	n/a	5 mL
Total	n/a	15 mL

**Laboratory supplies**

1. 1.5 mL glass vials with screw top lid (Sigma-Aldrich, catalog number: 854171)
2. Pierce Concentrator, PES membrane, 10K MWCO 0.5 mL (Thermo Fisher, catalog number: 88513)
3. Spin-X^R^ UF 20 10K MWCO PES membrane 20 mL (Corning, catalog number: 431488)
4. Amicon Ultra-15 centrifugal filters regenerated cellulose membrane 3K 20 mL (Millipore Sigma, catalog number: UFC901024)
5. NMR tubes (New Era Enterprises, Inc., catalog number: NE-UL5-7)
6. 1.5 mL microcentrifuge tubes (Cell treat, catalog number: 229441)
7. 20 mL scintillation vials (Wheaton, catalog number: 03-341-25K)
8. Pipettes (Corning, catalog numbers: 4075, 4071, 4072, 4074)
9. Pipette tips (Fisherbrand/Olympus, catalog numbers: 02-717-134/22-119B, 24-150RL, 23-404)
10. Glass pipettes (Fisherbrand, catalog number: 13-678-6B)
11. Separation funnel (Chem Glass, catalog number: CG1743-09)
12. 25 mL glass graduated cylinder (Chem Glass, catalog number: CG-8242-25)
13. Stir bar (Fisher Scientific, catalog number: 16-800-507)
14. Pipette bulbs (Fisherbrand, catalog number: 03-448-21)
15. Teflon tape (SPBel-Art, catalog number: 240200000)
16. Parafilm (Bemis Parafilm, catalog number: PM996)

## Equipment

1. Pioneer analytical balance (Ohaus, model number: PX84/E)
2. Navigator analytical scale (Ohaus, model number: NV222)
3. Eppendorf centrifuge 5425 (Eppendorf, catalog number: 5405000646)
4. Eppendorf thermomixer (Eppendorf)
*Note: The model used in this protocol was discontinued; a comparable model would be the Eppendorf Thermomixer, catalog number: 5384000020.*
5. LSE digital dry bath (Corning, catalog number: 6875-SB)
6. Hereaus megafuge 8 centrifuge (Thermo Scientific, catalog number: 75007210)
7. ST plus series centrifuge (Sorvall, catalog number: 75009909)
8. Speed vacuum concentrator (Savant, catalog number: SVC100H)
9. nX_DS_ vacuum pump (Edwards, catalog number: A73501983)
10. 4 °C refrigerator (VWR, catalog number: 10819-904)
-80 °C freezer (Fisherbrand, catalog number: IUE30086FA)
12. Zetasizer nano (Malvern Panalytical, model: ZEN3600)
13. 2800 ultrasonic bath (Branson, catalog number: M2800)
14. LSE vortex mixer (Corning, catalog number: 6778)
15. Mini centrifuge (Corning, catalog number: 6770)
16. AG centrifuge (Eppendorf, catalog number: 022620100)
17. Laboratory fume hood (Hamilton Laboratory Solutions, model: 61L)
18. Advance stir plate (VWR, catalog number: 76557-502)
19. Genesys 150 UV spectrophotometer (Thermo Scientific, catalog number: 840-300000)
20. pH meter (Oakton, catalog number: EW-35413-20)
21. High-field (≥ 500 MHz) NMR spectrometer, equipped with triple-resonance inverse probe

## Software and datasets

1. Prism v10.2 (GraphPad, 02/5/2024)
2. NMRPipe (v11.4) [11]
3. NMRFAM-Sparky (v3.12, 04/15/2015)
4. Bruker Topspin (v4.1.3)

## Procedure

- **Reverse micelle calculations**
  1. Determine the desired surfactant concentration.
*Note: This concentration may be tested for the protein of interest and the specific experiments to be performed. For the protein used here, GPx4 (18.6 kDa), a 75 mM surfactant concentration is used, while another common concentration is 150 mM. The concentration of 75 mM provides slightly better spectroscopic properties in the form of slightly narrower NMR line widths, while 150 mM allows for a higher protein concentration due to the increased aqueous phase volume.*
  2. Calculate the mass of surfactants needed for the desired ratio based on molecular weight. For a 50:50 molar percent ratio of natural lipid (or DLPC) to DPC:
    DPC (MW: 351.462): 6.6 mg
    DLPC (MW: 621.826): 11.6 mg
    or
    Soy lecithin: 12.1 mg
    or
    Porcine brain lipids: 12.1 mg
    or
    Bovine heart lipids: 12.1 mg
*Note: All compositions can form RMs at a 50:50 molar percent ratio of natural lipid (or DLPC) to DPC, but DLPC:DPC and lecithin:DPC work well up to 70:30 molar percent ratios. Soy lecithin, porcine brain, and bovine heart lipids are complex lipid mixtures, and the molar percent ratios are estimated as outlined in Walters et al. [4]*.
  3. Calculate the volume of water phase needed in relation to the total volume of the sample (500 μL) and the target water loading (W_0_). W_0_ is the molar ratio of water to surfactant.
  4.
$$W_{0}=\frac{\left[H_{2}O\right]}{\left[surfactants\right]}$$
    W_0_ = 20: 13.5 μL
*Note: W_0_ values differ based on the size of encapsulated protein, desired water dynamics, and target RM size. GPx4 encapsulates at W_0_ equal to and greater than 20, while ubiquitin (8.6 kDa) can be encapsulated at a W_0_ as low as 10. The range of 10–25 is typical in these systems.*
- **Protein preparation: concentration**
  There are two ways to prepare the protein for encapsulation: concentration or vacuum concentration/lyophilization. Each has its advantages and disadvantages. Every protein should be prepared both ways initially to determine the best method. Concentration is a faster process, and the protein can be encapsulated the day of, but it is more likely to aggregate during concentration.
  1. Use an appropriate 1.5 mL spin concentrator.
    In this example, for GPx4, a 10K MWCO PES membrane is appropriate.
  2. Verify if the initial protein concentration is ~1.2 mg/mL or higher through Bradford assay. Immediately before encapsulation, concentrate protein to the desired volume based on the W_0_.
*Note: Some proteins may survive concentrating at room temperature and others may need to be at 4 °C. This will be protein-dependent and will need to be evaluated for each protein.*
- **Protein preparation: vacuum concentration**
  If concentration does not result in optimal encapsulation, vacuum concentration (or lyophilization) results in higher encapsulation efficacy for many proteins but has to be prepared the day before sample preparation.
  1. The day before RM sample preparation, use a PES spin concentrator (10K MWCO, 20 mL) and buffer-exchange the protein sample from the original buffer to dilute buffer.
    - Prepare 60 mL of dilute protein buffer.
    - Add protein samples at 1.2 mg/mL (initial volume is 1 mL) and concentrate to 500 μL by centrifuging at 3,260× *g* until 500 μL is reached.
*Note: Some protein may be lost throughout the buffer exchange process. To account for this loss, up to 2-fold more protein is needed before concentration. Protein concentration may be verified through a Bradford assay after exchange.*
  2. Add dilute protein buffer to protein sample up to 20 mL and centrifuge until the volume is at 500 μL.
  3. Repeat two more times.
  4. Add a final 500 μL of protein volume to a 1.5 mL glass vial.
  5. Freeze protein solution for at least 10 min in a -80 °C freezer.
  6. Add vial to speed vacuum concentrator and allow to sublimate overnight, leaving only protein and dilute buffer components.
- **Soy lecithin metal contamination removal**
  This step is important when using soy lecithin to encapsulate protein in RMs. Without this step, the excess metal in the lecithin will induce line broadening in the ^31^P spectrum and may impact protein spectra (Supplementary Figure S2 [4]). Perform this step in a fume hood.
  1. Dry chloroform and methanol overnight with sodium sulfate. Decant the sodium sulfate via gravity filtration from the solvents the following day.
  2. Make 15 mL of mobile phase.
  3. Weigh 1 g of soy lecithin into a 20 mL scintillation vial.
  4. Add the mobile phase to the lecithin and allow to stir on a stir plate until dissolved.
  5. Add 5 mL of 200 mM EDTA pH 6.0.
  6. Allow to chelate by stirring for 24 h with a stir bar on a stir plate with the lid and parafilm.
  7. Add contents to a separatory funnel.
  8. Shake the funnel and release pressure as needed by slightly opening the spigot of the separatory funnel. Shake until fully mixed.
  9. Allow contents to separate; then, remove the bottom organic layer into a graduated cylinder.
  10. Add additional mobile phase up to 15 mL and transfer to a clean 20 mL scintillation vial.
  11. Add 5 mL of 200 mM EDTA pH 6.0.
  12. Allow to stir overnight.
  13. Repeat steps D6–8.
  14. Transfer the organic layer into a pre-weighed 20 mL scintillation vial and allow it to sit open to evaporate off the solvents overnight on a 37 °C heat block.
*Note: Dry lecithin overnight in a speed vacuum concentrator before use to remove excess moisture. The lecithin will be slightly oily and tacky.*
  15. To verify heavy metal extraction, add 75 mM lecithin (24.1 mg) to 500 μL of pentane and 13.5 μL of distilled water (W_0_ = 20). Titrate 1-hexanol in 50 mM increments (3.125 μL of 8 M 1-hexanol) until visual clarity is reached.
  16. Perform ^31^P 1D NMR scan to verify narrow lines and seven individual peaks corresponding to the different headgroup types using trimethyl phosphate as the ^31^P standard.
- **DLPC:DPC RM**
  1. The night before experimentation, place all surfactants in the speed vacuum concentrator to remove excess moisture.
  2. Weigh out surfactants based on the decided ratio into a 1.5 mL glass vial. For a 50:50 molar percent ratio DLPC:DPC RM, measure out 11.6 mg of DLPC and 6.6 mg of DPC.
  3. Add 500 μL of pentane to surfactants and vortex.
  4. Add 200 mM 1-hexanol (12.5 μL of 8 M 1-hexanol) to the solution and vortex until most solids are in solution.
  5. Add the desired water volume, including protein if needed.
    - If concentrating the protein, add directly from the spin concentrator to the solution.
    - If adding from vacuum-concentrated or lyophilized protein, add protein buffer to the protein and vortex until the protein is mostly in solution. Then, add the surfactants and solvent mixture to the vial containing the protein solution and vortex.
  6. Titrate and vortex in between each addition with additional 1-hexanol in 200 mM increments (12.5 μL of 8 M 1-hexanol) until 800 mM total 1-hexanol is reached; then, titrate with additional 50 mM 1-hexanol increments (3.125 μL of 8 M 1-hexanol). Titrate until there is a visually clear solution and then add an additional 50 mM 1-hexanol.
*Notes:*
    - *If adding from vacuum concentrated or lyophilized protein, the solution will need to be transferred twice (once into the original surfactant vial and once back into the protein vial) after the first and second additions of additional 1-hexanol from this step. This ensures that all surfactants and proteins are solubilized into the reverse micelle sample.*
    - *The typical needed range for 1-hexanol is 800 mM to 1.2 M, varying between different proteins and different sample conditions.*
**Pause point:** Once visual clarity is reached, the RM will remain stable for an extended period of time (up to several months for most stable proteins). When preparing samples a day ahead, it is recommended that, after formation, they be left on a thermomixer at room temperature until needed. Samples may also be stored in refrigerated conditions to prolong viability.
- **Lecthin:DPC RM**
  1. The night before experimentation, place all surfactants in the speed vacuum concentrator to remove excess moisture.
  2. Weigh out surfactants based on the decided ratio into a 1.5 mL glass vial.
    - For a 50:50 lecithin:DPC RM, measure out 12.1 mg of lecithin and 6.6 mg of DPC.
    - For a 70:30 lecithin:DPC RM, measure out 16.9 mg of lecithin and 3.95 mg of DPC.
  3. Add 500 μL of pentane to surfactants and vortex.
  4. Add 50 mM 1-hexanol (3.125 μL of 8 M 1-hexanol) to the solution and vortex until most solids are in solution.
  5. Add the desired water volume, including protein if needed.
    - If concentrating the protein, add directly from the spin concentrator to the solution.
    - If adding from vacuum-concentrated or lyophilized protein, add protein buffer to the protein and vortex until the protein is solubilized. Then, add the surfactants and solvent mixture to the vial with protein and vortex.
  6. Titrate with additional 1-hexanol in 50 mM increments (3.125 μL of 8 M 1-hexanol), vortexing in between each addition until there is a visually clear solution. Add an additional 50 mM 1-hexanol.
*Notes:*
    - *If adding from vacuum-concentrated or lyophilized protein, the solution will need to be transferred twice (once into the original surfactant vial and once back into the protein vial) after the first and second additions of additional 1-hexanol from this step. This ensures that all surfactants and proteins are solubilized into the reverse micelle sample.*
    - *The typical range needed for 1-hexanol is 400–700 mM, which varies according to the protein and sample conditions used.*
Figure 1 demonstrates the formation of lecithin:DPC nRMs. There is a need for proper addition of hexanol as well as a proper W_0_. The W_0_ must be carefully determined; if too little volume is added, the nRM will not form, and if too much volume is added, the solution will phase-separate (Figure 1A). If an insufficient amount of hexanol is titrated into the nRMs, visual clarity will not be reached and the nRMs will not form (Figure 1B). With the proper amount of hexanol, surfactants, and W_0_, a visually transparent solution will be acquired with fully formed nRMs (Figure 1C).
  **Figure 1.**
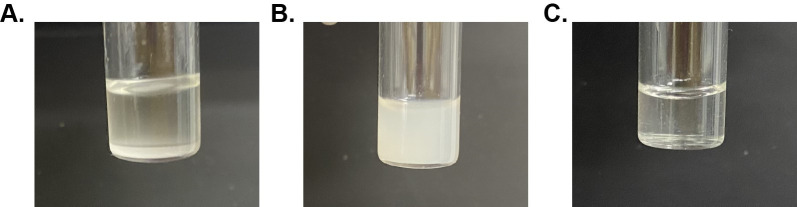
Two examples of empty native reverse micelle (nRM) formulations that do not solubilize and a fully solubilized nRM sample. A. 75 mM lecithin:DPC nRM with a W_0_ = 100 and 1 M hexanol displays a water content that is too large for nRM formation. Phase separation occurs and is observable by the separated aqueous layer at the bottom of the vial. B. 75 mM lecithin:DPC nRM with a W_0_ = 20 and 600 mM hexanol shows a hexanol concentration that is too low. nRM is not visually transparent; encapsulation and formation of nRMs is not complete, often remedied by the addition of more hexanol. C. 75 mM lecithin:DPC nRM with a W_0_ = 20 and 1 M hexanol. nRM is visually transparent; this indicates complete formation of nRMs.
- **PBL:DPC and BHL:DPC RMs**
  The PBL:DPC and BHL:DPC RMs can be formed with the same protocol. We describe the protocol for a PBL:DPC RM in this section, but the PBL can be substituted for the BHL with no additional alterations to the protocol.
  1. The night before experimentation, place all surfactants in the speed vacuum concentrator to remove excess moisture.
  2. Weigh out DPC based on the decided ratio into a 1.5 mL glass vial.
    For a 50:50 molar percent ratio PBL:DPC RM, measure out 6.6 mg of DPC.
  3. Make 100 mg/mL stock of PBL in pentane and calculate the needed PBL for 50:50 molar percent ratio.
  4. For this ratio, 22.5 mg/mL is needed, which is equal to 112 μL of PBL solution.
  5. Add 388 μL of pentane to DPC and vortex.
  6. Add 112 μL of the PBL/pentane solution to the vial and vortex.
  7. Add 50 mM 1-hexanol (3.125 μL of 8 M 1-hexanol) to the solution and vortex until most solids are in solution.
  8. Add the desired water content and protein as needed.
    - If concentrating the protein, add directly from the spin concentrator to the solution.
    - If adding from vacuum-concentrated or lyophilized protein, add water to the protein and vortex until the protein is mostly in solution. Then, add the surfactants and solvent mixture to the vial with protein and vortex.
  9. Titrate with additional 1-hexanol in 50 mM increments (3.125 μL of 8 M 1-hexanol), vortexing in between each addition until there is a visually clear solution. Add an additional 50 mM of 1-hexanol.
*Notes:*
    - *If adding from vacuum-concentrated or lyophilized protein, the solution will need to be transferred twice (once into the original surfactant vial and once back into the protein vial) after the first and second additions of additional 1-hexanol from this step. This ensures that all surfactants and proteins are solubilized.*
    - *Optionally, PBL solution may be added to the glass vial the night before and the solvent dried off with nitrogen and then in a speed vacuum concentrator overnight; then, DPC should be added directly into the glass vial with PBL. This will accommodate 500 μL of pentane being added after the lipids and step G4 may be omitted.*
- **NMR sample preparation**
  1. Add 10% deuterated solvent as a lock solvent to reverse micelles. For RM with pentane as the solvent, add 50 μL of d-pentane.
  2. If needed, deuterated alkane and surfactant may be used to suppress background signal and suppress artifacts for protein NMR experiments. Deuteration generally does not affect the stability of RM systems. However, at this time, deuterated lipid extracts are not available.
  3. Add the sample into an NMR tube and cover with Teflon tape to reduce evaporation of solvents.
*Note: Screw-top NMR tubes, such as Norell, catalog number: C-S-5-600-SC-7, may be used to mitigate evaporation in longer 3D NMR experiments. However, in our experience, a well-sealed standard NMR tube can be used in most cases.*
- **NMR experiments**
  1. Add 10% deuterated solvent as a lock solvent to the reverse micelle sample.
    - For RMs with pentane as the solvent, add 50 μL of d-pentane or d-hexane.
    - The pentane (or hexane) methyl signal is used for the NMR spectrometer lock.
  2. Standard Bruker pulse sequences are suitable for the approach reported here.
  3. Echo-anti echo gradient–selected NMR experiments are typically used for suppression of solvent and lipid/surfactant signals.
  4. Water suppression using flip-back pulses is generally not necessary and may be turned off due to the low overall concentration of water in the RM system.
  5. Pulse length and water frequency are calibrated as typical for aqueous NMR samples.
  6. ^15^N-HSQC experiments of encapsulated proteins are typically collected to confirm encapsulation and map membrane interactions of PMPs. The first increment of ^15^N-HSQC experiments may be used to track sample stability over time (Figure 2). 2D-^15^N-HSQC experiments provide amino acid resolution, which may be compared to a reference through calculated chemical shift perturbations (CSPs).
    **Figure 2.**
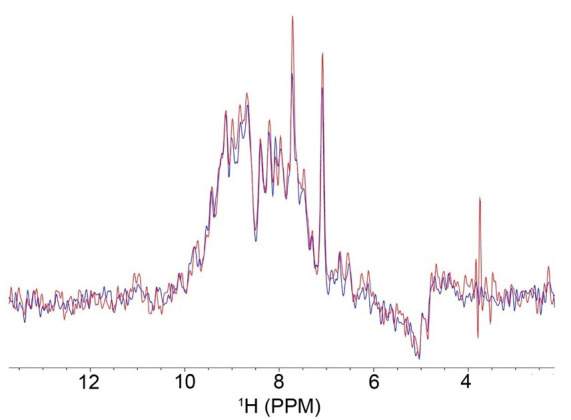
Overlay of the first increment of ^15^N-HSQC experiments of GPx4 in a 50:50 molar percent ratio lecithin:DPC native reverse micelle (nRM) with 100 μM of GPx4 in nuclear magnetic resonance (NMR) buffer with 1 M hexanol, collected multiple days apart to check sample stability. The red spectrum was collected on day one, and the blue spectrum was collected on day 4, with the sample stored at room temperature throughout. The overlay indicates that very little protein signal was lost between experiments, confirming protein stability within the nRM.
- **DLS experiments**
  1. Collect DLS measurements in a quartz cuvette at room temperature. A typical sample volume is 500 μL. Hexane may be used for DLS, since signal loss from slow tumbling is not a concern and hexane evaporates more slowly than pentane.
  2. Use published viscosity and dielectric constant parameters using the volume ratio of hexane to hexanol [12,13].
    A typical ratio used for the determination of the viscosity and dielectric constant for a nRM with 1 M hexanol in hexane is 0.875. The viscosity value using these parameters is 0.356 and the dielectric constant is 2.10. The refractive index value is kept constant at 1.38.
  3. Data were collected on a Malvern Zetasizer nano using standard DLS measurement settings.
  4. DLS measurements are typically collected in triplicate to calculate error. Figure 3 demonstrates the DLS measurements of GPx4 encapsulated in a lecithin:DPC nRM.
    **Figure 3.**
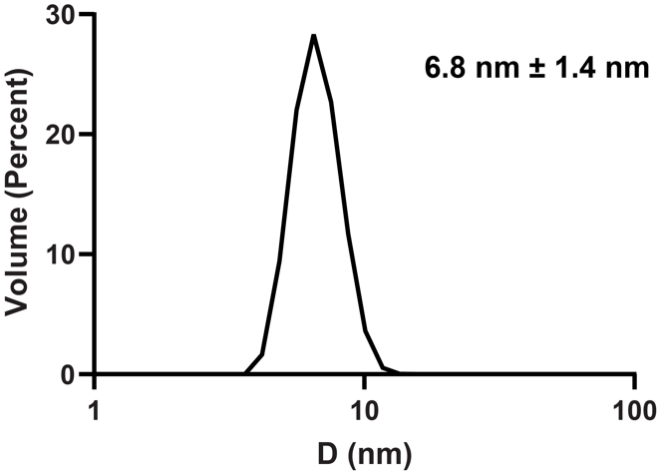
Dynamic light scattering (DLS) size distribution of 75 mM lecithin:DPC nRM with a W_0_ = 20 encapsulating 100 μM GPx4 in 20 mM Bis-Tris pH 6.0, 100 mM NaCl, and 10 mM DTT, with 1 M hexanol as the cosurfactant. Average diameter is 6.8 nm with a size distribution of 1.4 nm.

## Data analysis

The analysis of the protein NMR spectra of encapsulated PMPs has been previously described in Labrecque et al. [5]. Briefly, all NMR data were processed using the NMRPipe software and visualized and analyzed using the NMRFam-Sparky distribution. The assignments for aqueous GPx4 have been previously published (BMRB: 50955) and the encapsulated protein assignments were transferred from the aqueous assignments as described in Labrecque et al. [5]. The chemical shift perturbations were calculated using the following formula using weighted shifts:

$$CSP=\sqrt{({∆}^{1}H{)}^{2}+{\left(\frac{{∆}^{15}N}{9.8655}\right)}^{2}}$$

**Figure 4.**
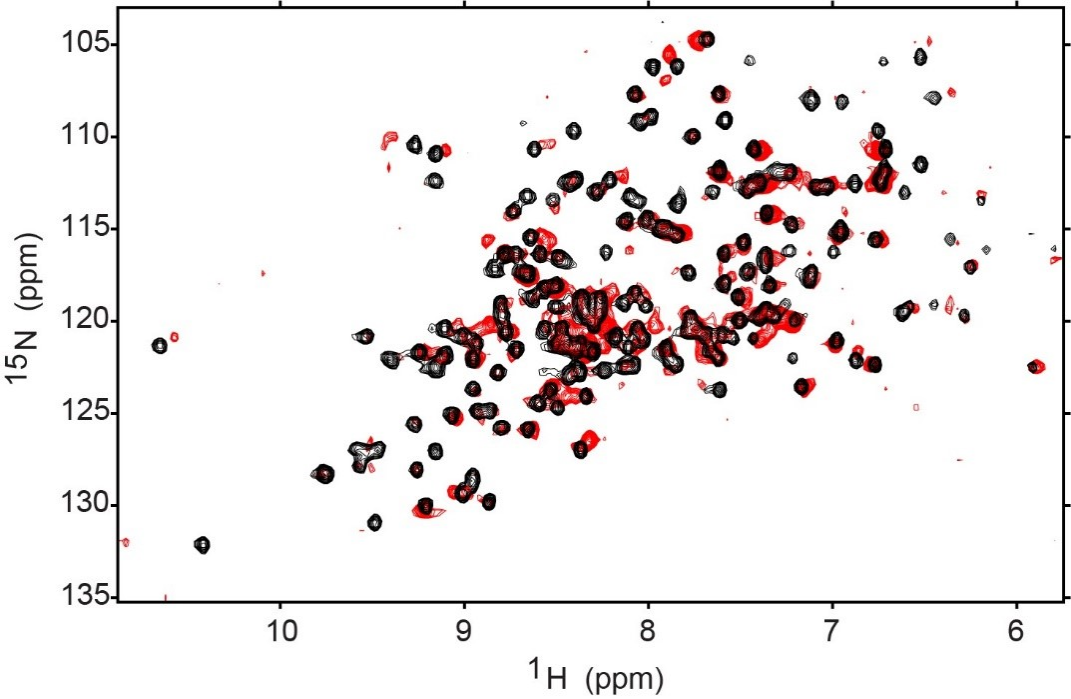
^1^H-^15^N HSQC of aqueous GPx4 (black) and encapsulated GPx4 (red). The encapsulation conditions are 75 mM 50:50 molar percent ratio lecithin:DPC with 1 M hexanol (GPx4 buffer: 20 mM Bis-Tris pH 6.0, 100 mM NaCl, and 10 mM DTT). Both HSQCs were collected at 25 °C and 600 MHz on a room temperature probe and processed on NMRPipe and the NMRFAM-Sparky distribution. Contours were lowered to near the noise level in both spectra.

∆^1^H and ∆^15^N represent the changes in the ^1^H and the ^15^N chemical shifts for each resonance. Resonance CSPs can be mapped to the corresponding residues on the structure of the protein for a view of membrane interaction surfaces. Figure 4 demonstrates the shifting that occurs when GPx4 is encapsulated in a nRM compared to its aqueous state.

## Validation of protocol

The protocol described here has been used and validated in:

- Walters et al. [4]. Investigating protein-membrane interactions using native reverse micelles constructed from naturally sourced lipids. *Protein Science* (Figure 2, panels a–f) (Figure 4, panels a, d) (Figure 5, panels c, f, i) (Supplementary Figure 2, panels a–e).
- Labrecque et al. [5]. Membrane-Mimicking Reverse Micelles for High-Resolution Interfacial Study of Proteins and Membranes. *Langmuir* (Figure 4, panels a–b) (Supplementary Figure 2, panel a).

Successful encapsulation of GPx4, along with other proteins, is shown in Walters et al. [4] and Labrecque et al. [5] for all RM systems described here. The confirmation of encapsulation was determined via protein NMR and the validity of the compositions was confirmed through comparable chemical shift perturbation (CSP) in the active and binding sites of the proteins. Ubiquitin was used as a control protein, and NMR revealed that the formulations of the reverse micelles did not impact the aqueous structure of ubiquitin. DLS confirms the formation of uniformly sized RMs, which increase in diameter as the water loading is increased, as expected.

## General notes and troubleshooting

**General notes**

1. Pentane is a highly volatile solvent, which makes it difficult to maintain the proper volume. Pipette carefully with either a glass pipette or a micropipette by slowly bringing the solvent into the pipette, and then maintaining exact pressure between vials before ejecting it into the new vial.
2. Alternatively, hexane may be used as the solvent. This mitigates evaporation but results in more NMR line-broadening for larger protein systems.
3. Protein stability can be a concern during the formation of the reverse micelles due to the high concentration in the water phase before addition to the surfactant/solvent phase. If protein aggregates, remove aggregate before addition to reverse micelle. If protein concentration is lost during buffer exchange, add additional protein before exchanging to mitigate loss.
4. If possible, NMR spectra of the protein of interest should be collected in membrane models with little or no curvature, such as isotropic bicelles or nanodiscs, and compared to a spectrum of the protein within nRMs. Major differences in spectra may indicate curvature-dependent conformational differences, and caution is warranted. Smaller spectral differences are likely indicative of differences in lipid content among the membrane models and suggest that the nRM is an adequate model for the protein of interest.
5. Alkanes and hexanol may bind to or otherwise perturb the proteins of interest. The above-described control using either isotropic bicelles or nanodiscs may also reveal changes in the protein structure from the solvent components. Due to its high water solubility, hexanol may be added to aqueous samples to determine if it is a strong binder to the protein of interest or if it otherwise perturbs the protein structure.

**Troubleshooting**

Problem 1: Reverse micelle is not reaching visual clarity.

Possible causes: Excess of lipids or protein or non-optimized water loading volume.

Solution: Verify surfactant and protein concentrations. Allow to shake for 1 h or sonicate in a water bath for 15 min. Slowly add additional 1-hexanol titrations. Adjust the water loading value (increase). In some cases, the reverse micelle will not fully clear and the additional hexanol will not improve the visible state of the reverse micelle. If this is the case, test the sample in the NMR for protein signal. Some proteins and sample conditions will produce high-quality NMR spectra without complete visual clarity.

Problem 2: Protein does not encapsulate efficiently, aggregation during concentration.

Possible cause: Sub-optimal stability in the high concentration necessary for delivering concentrated protein.

Solution: If the protein is amenable to vacuum concentration or lyophilization: vacuum concentrate or lyophilize the protein in a glass vial and construct the reverse micelle without the protein. Allow the reverse micelle to form without protein; then, after visual clarity is reached, add reverse micelle sample to the dried protein and vortex. Allow to shake overnight if the addition of protein does not immediately lead to a clear solution. If reverse micelle does not clear by the next day, titrate an additional 1-hexanol in 50 mM increments until visual clarity is reached or high-quality protein NMR spectra are observed.

Problem 3: Reverse micelle sample volume decreased over time.

Possible cause: Pentane is volatile and will rapidly evaporate.

Solution: Leave vials with pentane sealed when not actively adding to the vial. Once the reverse micelle is formed, add additional pentane up to 500 μL and seal the cap with Teflon tape.
